# Seasonality of Influenza and Respiratory Syncytial Viruses and the Effect of Climate Factors in Subtropical–Tropical Asia Using Influenza-Like Illness Surveillance Data, 2010 –2012

**DOI:** 10.1371/journal.pone.0167712

**Published:** 2016-12-21

**Authors:** Taro Kamigaki, Liling Chaw, Alvin G. Tan, Raita Tamaki, Portia P. Alday, Jenaline B. Javier, Remigio M. Olveda, Hitoshi Oshitani, Veronica L. Tallo

**Affiliations:** 1 Department of Virology, Tohoku University Graduate School of Medicine, Sendai, Japan; 2 Department of Epidemiology and Biostatistics, Research Institute for Tropical Medicine, Department of Health, Manila, Philippines; 3 Research Institute for Tropical Medicine, Department of Health, Manila, Philippines; Kliniken der Stadt Köln gGmbH, GERMANY

## Abstract

**Introduction:**

The seasonality of influenza and respiratory syncytial virus (RSV) is well known, and many analyses have been conducted in temperate countries; however, this is still not well understood in tropical countries. Previous studies suggest that climate factors are involved in the seasonality of these viruses. However, the extent of the effect of each climate variable is yet to be defined.

**Materials and Methods:**

We investigated the pattern of seasonality and the effect of climate variables on influenza and RSV at three sites of different latitudes: the Eastern Visayas region and Baguio City in the Philippines, and Okinawa Prefecture in Japan. Wavelet analysis and the dynamic linear regression model were applied. Climate variables used in the analysis included mean temperature, relative and specific humidity, precipitation, and number of rainy days. The Akaike Information Criterion estimated in each model was used to test the improvement of fit in comparison with the baseline model.

**Results:**

At all three study sites, annual seasonal peaks were observed in influenza A and RSV; peaks were unclear for influenza B. Ranges of climate variables at the two Philippine sites were narrower and mean variables were significantly different among the three sites. Whereas all climate variables except the number of rainy days improved model fit to the local trend model, their contributions were modest. Mean temperature and specific humidity were positively associated with influenza and RSV at the Philippine sites and negatively associated with influenza A in Okinawa. Precipitation also improved model fit for influenza and RSV at both Philippine sites, except for the influenza A model in the Eastern Visayas.

**Conclusions:**

Annual seasonal peaks were observed for influenza A and RSV but were less clear for influenza B at all three study sites. Including additional data from subsequent more years would help to ascertain these findings. Annual amplitude and variation in climate variables are more important than their absolute values for determining their effect on the seasonality of influenza and RSV.

## Introduction

The seasonality of influenza [1] and respiratory syncytial virus (RSV) [2] is a phenomenon that has been long known but not well understood. In contrast to the clear seasonal patterns observed in temperate countries, less defined influenza peaks have been reported in tropical Asia, ranging from biannual peaks [3–5] to low-level circulation throughout the year [6, 7]. Climate factors are known to play an important role; however, their contribution to observed seasonality patterns is yet to be defined [8].

Recent studies analyzed the seasonal variations of influenza [9, 10] and RSV [11, 12] at a global scale, by pooling available data from various countries. Seasonal influenza epidemics in tropical and temperate areas were observed during humid–rainy and cold–dry seasons, respectively [9]. When compared with countries at high or low latitudes, those at intermediate latitudes showed less correlation between the timing of influenza peaks and climate variables. This intriguing phenomenon could be explained by a possible U-shaped relationship between humidity and influenza virus viability, as proposed in animal experimental studies [8, 13, 14]. However, this hypothesis remains controversial as other studies reported an inverse relationship [15, 16]. For RSV, associations between epidemics and climate variables tend to vary across time and geographic locations [12], with less consistency in tropical regions [17].

Ascertaining the role of climate factors is difficult due to their involvement in a number of mechanisms leading to host-to-host transmission, including host immunity, indoor host contact, and virus survival in the environment [18]. For the latter, the effects of climate factors have been studied in animal experiments [19], cough simulations [20], and its effect on virus structure [21]. Despite both being enveloped RNA viruses with generally similar transmission routes, differences exist between the seasonality of influenza and RSV. Both viruses can be transmitted by any of the following modes: airborne, droplet, or contact transmission. However, the significance of each route for influenza [22, 23] and RSV [24, 25] varies, depending on the setting and context. Greater understanding of the climate factors affecting influenza and RSV would thus help to explain the major modes of transmission, particularly at low and intermediate latitude areas. Also, more understanding on the climate effects on influenza activity in these areas will also contribute information on the optimal timing of influenza vaccination.

In this study, we attempted to address these issues by analyzing the surveillance datasets of influenza virus and RSV. The primary objective of this study is to determine the association between climate variables and the detection of influenza A, influenza B, and RSV at three Asian sites located at low and intermediate latitudes: the Eastern Visayas region and the city of Baguio in the Philippines, and Okinawa Prefecture in Japan. These sites were chosen to investigate any differences owing to changes in latitude (all three sites) and altitude (both Philippines sites). We also investigated the seasonal patterns of influenza and RSV in tropical and subtropical Asia (S1 Table and S2 Table).

## Results

### Patterns of circulating viruses at the three study sites

Table 1 summarizes the tested influenza-like illness (ILI) samples and all virus positives at the three sites during the study period. The total number of nasopharyngeal samples collected in Baguio City (BC) was about 2.7 times higher than that in Eastern Visayas (EV). The number of influenza A positive samples was higher than that positive for influenza B at all three sites, except for EV and BC in 2010. Both EV and BC had respectively 2.0 and 1.4 times more RSV positive cases than those for overall influenza. Data on the number of samples collected were unavailable for Okinawa (OK).

**Table 1:** Summary of the total annual samples tested and positive cases of influenza A, influenza B, and RSV detected at the three study sites, from January 2010 to December 2012.^a^

		2010	2011	2012	Total
**Eastern Visayas**	Total samples	605	645	655	1905
	Flu A +	54 (8.9)	55 (8.5)	18 (2.8)	127 (6.7)
	Flu B +	61 (10.1)	35 (5.4)	7 (1.1)	103 (5.4)
	RSV +	55 (9.1)	93 (14.4)	63 (9.6)	211 (11.1)
**Baguio**	Total samples	2022	1737	1477	5236
	Flu A +	214 (10.6)	168 (9.7)	158 (10.7)	540 (10.3)
	Flu B +	317 (15.7)	49 (2.8)	81 (5.5)	447 (8.5)
	RSV +	304 (15.0)	186 (10.7)	136 (9.2)	626 (12.0)
**Okinawa**	Total samples	N/A	N/A	N/A	N/A
	Flu A +	144	158	111	413
	Flu B +	9	44	52	105

^a^ Parentheses indicate the annual percentage positive for each virus. This was not calculated for Okinawa owing to lack of data on total samples tested.

**Fig 1:**
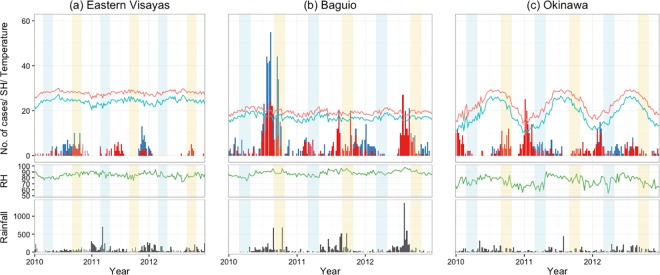
**Weekly climate trends and number of confirmed influenza A (red) and influenza B (blue) cases, detected in (a) Eastern Visayas, (b) Baguio and (c) Okinawa, from January 2010 to December 2012.** The black bars (bottom) indicate amount of rainfall and the lines indicate mean temperate (red), specific humidity (SH; blue), relative humidity (RH; green). Light blue and light yellow shaded areas represent the usual timing of influenza vaccination for the Southern (March—April) and Northern (September—October) hemispheres, respectively.

**Fig 2:**
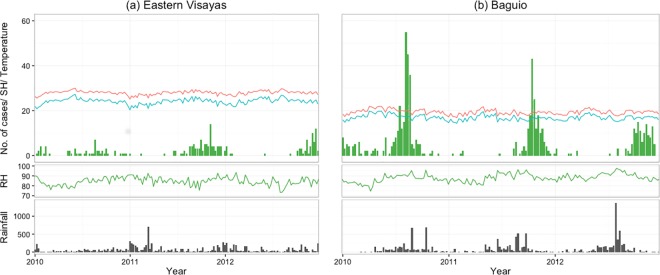
**Weekly climate trends and number of confirmed RSV cases detected in (a) Eastern Visayas, (b) Baguio from January 2010 to December 2012.** The black bars (bottom) indicate amount of rainfall and the lines indicate mean temperate (red), specific humidity (SH; blue), relative humidity (RH; green).

Fig 1 shows the weekly number of influenza positives detected at the three sites. Influenza A epidemics were observed in both winter and summer in Okinawa, although the number of influenza positives in summer was fairly small. Influenza B was detected in 36.5% of the total study weeks, with no clear epidemic pattern. In BC and EV, peaks of influenza positives were generally observed during the second half of the year. No influenza virus was detected for 19.9% and 55.8% of the total weeks in BC and EV, respectively. A three-week lag from EV to BC showed the highest cross-correlational coefficient (r^2^ = 0.24) for overall influenza. Fig 1 also included the timing of influenza vaccination for both hemispheres. Influenza activity was detected across the timing of vaccination for Northern hemisphere in BC and EV, except in 2012 while observed after the timing of for the Southern hemisphere. In OK, major peaks of influenza activity were detected after timing of vaccination timing for the Northern hemisphere. In the Philippines, no RSV was detected in EV and BC for 51.3% and 48% of the total weeks, respectively (Fig 2).

**Fig 3:**
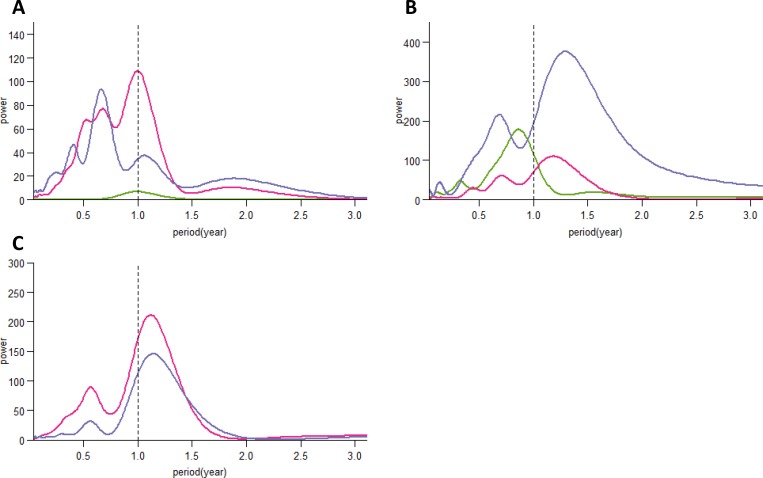
**Global wavelet power spectra for influenza A (A), influenza B (B), and RSV (C) positives at the three study sites.** Colours indicate study sites (Okinawa, green; Baguio, pink; Eastern Visayas, purple).

The global wavelet power spectra for influenza A showed consistent annual seasonality for OK and BC, whereas the highest power value was observed at a period of 0.7 in EV (Fig 3A). On the contrary, we observed less seasonal stationarity in the influenza B time series (Fig 3B). Only OK showed the highest power value at a period of 0.9; BC and EV showed power values at periods of 1.2 and 1.3, respectively. For RSV, both sites in the Philippines demonstrated seasonal stationarity (Fig 3C).

### Patterns of climate variables at the three sites

Figs 1 and 2 also show the trend of weekly climate variables at the three sites during the study period. OK had an annual cyclic pattern of climate variables, but the pattern was less clear for RH and precipitation. In contrast, both EV and BC had a less distinct cyclic pattern; the climate variables for both sites had narrower interquartile ranges than that of OK (S1 Fig). There were no significantly different distributions in mean temperature, RH, and SH between years, except for precipitation in BC and EV (*p*<0.001). The medians of mean temperature, RH, and SH were significantly different across the three sites (*p*<0.001); their interquartile ranges in OK were 7.9-, 2.2-, and 8.8-fold higher than the mean value from both Philippine sites, respectively. Owing to its high altitude, BC has lower mean temperature and SH than EV. The median amount of precipitation was significantly higher in EV than that in BC and OK (*p* = 0.03). Precipitation was concentrated in the midyear at both Philippine sites but was observed throughout the year in EV.

### Time series analysis of virus positives and climate variables

**Fig 4:**
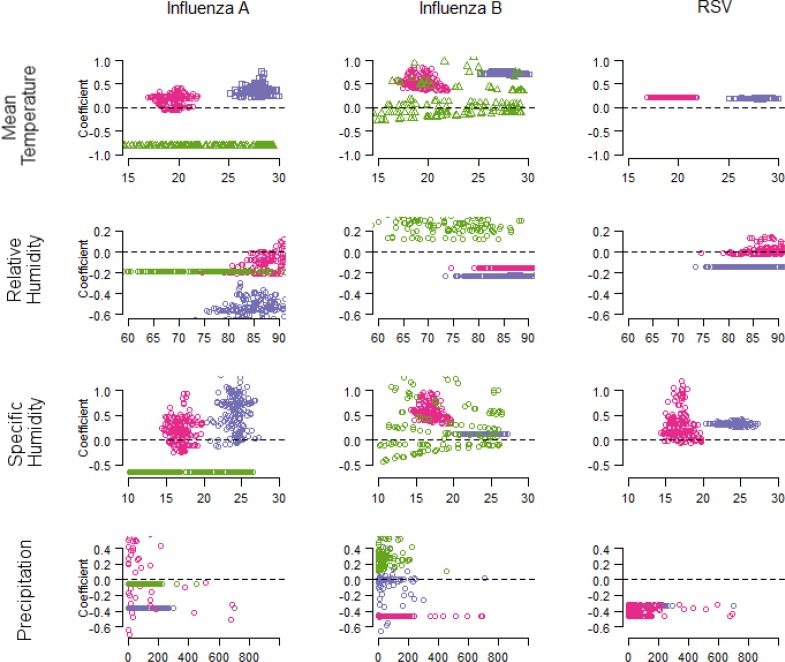
**Coefficients of climate variables estimated with state-space model for influenza A (left), influenza B (middle), and RSV (right).** Colours indicate study sites (Okinawa, green; Baguio, pink; Eastern Visayas, purple).

A dynamic linear regression model was applied to develop a time series model for virus positives (Table 2). All climate variables except rainy days improved model fit to the local trend model for both influenza (S3 Table) and RSV. The percentage improvement of model fit was higher among the models for OK than those for the Philippine sites. In OK, SH and mean temperature contributed substantial improvement to the model fit for influenza. Unlike influenza B, the coefficient for SH remained negative for influenza A (Fig 4). In the Philippines, the contribution of SH to model fit differed between sites and influenza types, however the model for the combination of influenza A and B indicated positive correlation and similar improvement to model fit (S2 Fig). Precipitation also improved model fit, particularly for influenza B in EV. The coefficients for precipitation remained negative for influenza A in EV and influenza B in BC. For RSV, precipitation contributed the highest improvement to model fit for both Philippine sites, followed by SH and mean temperature. Whereas the coefficient for precipitation was negative, those for the other variables were positive.

**Table 2:** Model performance for influenza A, influenza B and RSV to fit with climate variables in Eastern Visayas, Baguio, and Okinawa.

	Model	Eastern Visayas		Baguio		Okinawa	
		AIC	Improved model fit (%)	AIC	Improved model fit (%)	AIC	Improved model fit (%)
Flu A	Local trend	−819.2		−690.2		−678.3	
	Mean temperature	−811.6	0.9	−686.8	0.5	−645.0	4.9
	RH	−812.5	0.8	−684.2	0.9	−648.6	4.4
	SH	−811.1	1.0	−687.8	0.3	−646.1	4.8
	Rainfall	−814.2	0.6	−678.3	1.7	−650.1	4.2
	Rainy days ^a^	−863.3	-5.4	−658.8	4.5	−712.2	-5.0
Flu B	Trend	−970.3		−725.4		−839.1	
	Mean temperature	−962.2	0.8	−717.3	1.1	−809.7	3.5
	RH	−965.8	0.5	−722.1	0.5	−818.0	2.5
	SH	−965.5	0.5	−717.1	1.1	−797.2	5.0
	Rainfall	−916.0	5.6	−717.8	1.1	−827.3	1.4
	Rainy days ^a^	−916.0	5.6	−784.7	-8.1	−866.8	-3.3
RSV	Trend	−726.1		−715.9			
	Mean temperature	−722.5	0.5	−712.2	0.5		
	RH	−722.9	0.4	−714.3	0.2		
	SH	−721.3	0.7	−712.2	0.5		
	Rainfall	−720.8	0.7	−708.5	1.0		
	Rainy days ^a^	−771.5	-6.2	−745.3	-4.1		

Abbreviations: AIC: Akaike Information Criterion, RH: relative humidity, SH: specific humidity.

^a^ Rainy day is defined as the number of rainy days in one week with > 10 mm precipitation.

## Discussion

We analyzed influenza and RSV seasonality as well as investigated the driving role of climate variables at three Asian sites. An annual stationarity pattern was observed for influenza A and RSV, whereas influenza B showed a longer circulation period, particularly in the Philippines. No particular influenza or RSV epidemic was observed in the Philippines during the temperate winter period. The observed latitudinal differences for influenza activity are consistent with findings from countries that span several climate types [26–28].

We found that mean temperature as well as both specific and relative humidity in OK were inversely associated with influenza A activity, which is in line with observations in other temperate areas [29]. As proposed in both animal [19] and virus [30] experimental studies, dry atmospheric conditions may enhance the aerosol transmission route of influenza. Both minimum temperature and SH in OK fell below the threshold suggested by a pooled analysis [9] during winter, suggesting effective virus transmission under cold–dry conditions. Notably, climate variables in OK were more variable than those of the Philippine sites. This indicates that high annual amplitude could potentially offer a better explanation for the seasonality of infectious diseases. Influenza B was usually observed after the winter influenza A activity, as found in previous studies [31, 32]. Influenza B activity was still detected during the rainy season in OK, especially in 2011 and 2012. This could explain why we found a positive relationship between influenza B activity and both relative humidity and precipitation.

In the Philippines, there was a positive relationship between both influenza types and SH, which is consistent with data reported for tropical Vietnam [33] and South America [34]. This could be explained by the viability of influenza virus in airborne respiratory droplets [21, 30]. At high humidity, low evaporation of respiratory droplets allows the virus to maintain its viability in the environment [14]. These droplets then settle onto surfaces owing to gravity, thereby creating a reservoir for contact transmission [14]. In addition, humid conditions were previously suggested to be instead conducive for contact transmission in tropical settings [25, 35]. Thus, our results at both Philippine sites suggest an active role of contact transmission of both influenza A and B.

With influenza models for both Philippine sites, the coefficient for SH in the influenza B model in EV was 0.1 whereas that in BC ranged from 0.3 to 1.0. This suggests that the extent of the effect of SH on influenza B activity is less in hot–humid conditions, a phenomenon also documented in subtropical Hong Kong for RH [5]. The mechanism remains unclear; however, a low level of virus persistence observed at high temperatures [20] could minimize the effect of SH on influenza B activity.

In BC, the ranges of temperature and humidity fell outside those of hot–humid and cold–dry conditions suggested by the pooled analysis [9]. BC can instead be regarded to have cold–rainy conditions, mainly owing to its high altitude (1472m). Since most coefficient values (except for precipitation in the influenza A model) were comparable to those in EV, this suggests that the annual amplitude of climate variables has a greater role in explaining influenza seasonality than their absolute values.

We found an overlap between influenza activity in EV and BC and the timing of vaccination in Northern hemisphere. As a recent study [36] described, it is not sufficient to decide an optimal timing of influenza vaccination only based on a country's hemispheric position; it is also necessary to consider the local influenza activity trends.

RSV activity in the Philippines showed an annual seasonal pattern during the study period. First, less marked latitudinal differences were found for the RSV model when compared with that of influenza. There was a positive relationship between RSV activity and mean temperature in both EV and BC. This finding is compatible with those in other nearby countries [3, 37, 38], although an inverse association has also been reported in the Netherlands [39], and Malaysia [40, 41]. Second, precipitation showed an inverse relationship with RSV activity, a finding consistent with those in tropical Kolkata, India [42]. However, this finding is in contrast to those reported in tropical Southeast Asia [37, 40, 43] and Brazil [44].

We chose a state-space time series model to use the time dependency aspect of climate parameters. In addition to the role of humidity in influenza activity [8], the effects of other climate factors differed by both location and time. Furthermore, ranges of the climate variables were narrow, especially for EV and BC. Studies of meteorological effects on influenza activity mainly use regression methods [10] and/or time series methods [45, 46]. Implementing flexible parameterization could allow us to also consider the effect of climate variable with time.

We should note that climate variables added only modest improvement (0.2%–5.6%) to the local trend model. Determining the absolute value of this improvement was imprecise; however, we identified some underlying issues. First, aggregating the data at a weekly scale might affect the model. Because we were investigating the overall trend of virus activity, using data at a daily scale may yield a more accurate model. Second, the trend process itself can involve a stationarity process similar to climate variables. Third, climate variables may contribute a smaller fraction than other factors such as social behaviour and viral–human interaction in the environment. Further studies on social mixing in populations could allow us to identify other possible explanatory variables. Fourth, studies on the relationship between virus activity and indoor climate conditions are warranted. This is due to two reasons: (i) host-to-host transmission of influenza and RSV mainly occur indoors, and (ii) both absolute values and variance in the climate variables differ between outdoor and indoor settings.

This study has some limitations. First, this study covers a three-year study period, which could be considered short for an in-depth analysis. One reason for this is the logistical constraints encountered while collecting daily climate data from both Philippine sites. However, we were able to find temporal stationarity within the number of virus positives reported and also with climate factors. Second, the number of sentinel sites was different among the three sites; consequently the size of the catchment population was also different. To minimize this effect, we normalized both influenza and RSV positives prior to analysis. Third, we used cases confirmed by real-time polymerase chain reaction (RT-PCR) as the outcome variable in our study, resulting in potentially underestimating the true number of infected people. This limitation is especially true for OK because only a small proportion was tested by RT-PCR. Although the trend of influenza RT-PCR positives correlated well with that of point-of-care positives (S3 Fig), the latter could not detect a small peak in 2010. Fourth, climate data for OK was collected only for its capital city (Naha), which is located on the largest Okinawa Island. We think this is reasonable because the climate conditions on the main island where about 90% of the population live are similar to those on the other Okinawa Island.

In conclusion, annual seasonal circulation was observed for influenza A and RSV but less so for influenza B, in our three study sites. Mean temperature and SH showed a positive relationship with influenza A, B, and RSV at both Philippine sites whereas the inverse was found for influenza A in Okinawa. Rather than the values of climate factors, their annual amplitude and variation are more important to determining the effect of climate on the seasonality of influenza and RSV. Including additional data from subsequent more years would help to ascertain these findings.

## Materials and Methods

### Study sites and data collection

**Fig 5:**
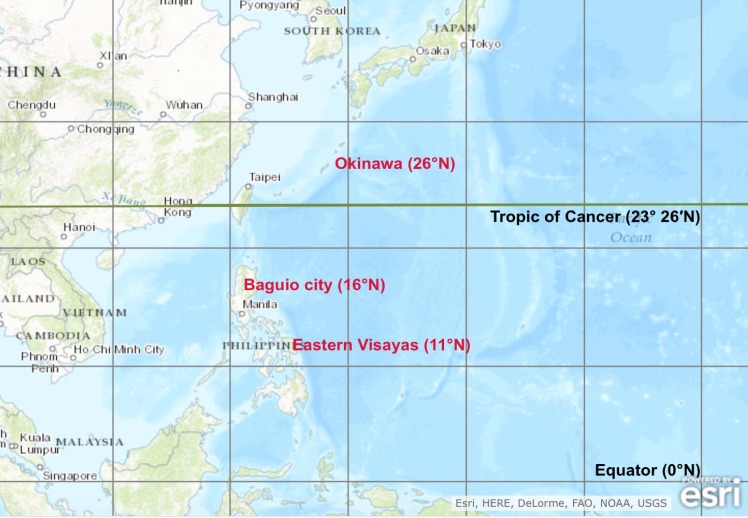
Geographic locations of the three study sites: Eastern Visayas and Baguio City (Philippines), and Okinawa Prefecture (Japan). Reprinted from https://upload.wikimedia.org/wikipedia/commons/4/48/Geographical_distribution_of_sites_in_the_Philippines_%28Eastern_Visayas_and_Baguio_city%29_and_Japan_%28Okinawa_Prefecture%29.tif, under a CC BY-SA 4.0 license, with permission from Liling Chaw, original copyright 2016.

Data were collected from influenza-like illness (ILI) surveillance programs conducted at three sites: the Eastern Visayas region (EV) and Baguio City (BC) in the Philippines, and Okinawa Prefecture (OK) in Japan (Fig 5), from January 2010 to December 2012 (S4 Table). Using the definition of tropical zone as ≤ 23.5° N/S [10, 11], both Philippine sites are situated in the tropical zone whereas the OK site is in the subtropical zone. Under the Köppen-Geiger climate classification [47], EV, BC, and OK are classified as tropical rainforest or equatorial, subtropical highland, and subtropical climates, respectively.

EV (11.2° N latitude, 125°E longitude) is located in the central eastern part of the Philippines. ILI surveillance was conducted at three public health facilities (Tacloban City Health Centre, Leyte Provincial Hospital, and Tanauan Rural Health Unit), as previously described [48]. BC (16.4°N latitude, 120.6°E longitude) is located in the northern region of the Philippines and is situated 1472 m above sea level. ILI surveillance was conducted at all 16 city health centres and outpatient departments in the government hospital [49]. Both sampling and testing protocols in EV and BC were compatible. Briefly, the total weekly number of ILI case counts were recorded and nasopharyngeal swabs were collected from patients on a regular basis. An ILI case was defined based on World Health Organization guideline [50]. Collected specimens were tested for influenza and RSV by RT-PCR, as previously described [48, 49].

OK (26.2°N latitude, 127.7°E longitude) is located in the southernmost part of Japan and consists of nearly 50 inhabited islands. About 90% of its population lives on the main island of Okinawa where its capital city, Naha, is located. ILI was defined as the sudden onset of fever, upper respiratory symptoms and/or general febrile symptoms, or as a case diagnosed as influenza by point-of-care testing. Selected samples were further tested by RT-PCR at a local public health laboratory. The weekly positive data were obtained from OK’s Infectious Disease Information Centre website [51]. ILI surveillance was conducted throughout the prefecture, with more than half of its sentinel sites located in Naha.

### Climate dataset and calculation

Daily data were compiled for the following climate variables during the same time period: temperature (mean, minimum, maximum), relative humidity (RH), mean atmospheric pressure, and precipitation. For EV and BC, available data were obtained from the local meteorological station [52]. Each station was chosen owing to its proximity to the ILI surveillance sites. Both stations were managed by the Philippine Atmospheric, Geophysical and Astronomical Services Administration, a national agency that provides meteorological and hydrological services for the country (the Philippine Atmospheric, Geophysical and Astronomical Services Administration). For OK, data for the city of Naha was obtained from the Japan Meteorological Agency website [53] (S4 Table).

Owing to data unavailability, some climate variables were derived using existing formulae. Specifically, mean atmospheric pressure in BC was calculated using the barometric formula [54], and specific humidity (SH) was calculated using both mixing ratio and SH formulae [55] (S1 Text). SH serves as a proxy measure for absolute humidity, which is defined as the mass of water vapour per unit volume of air. RH is defined as the ratio of actual water vapour pressure to the saturated vapour pressure of the air. We also calculated the weekly number of rainy days using daily precipitation data. A cut-off point of > 10 mm precipitation was set to define a rainy day.

### Statistical methods

We investigated both the autocorrelation and cross-correlation of weekly positive influenza data. The dataset was primarily tested for a unit root, then a 1 lag difference if the original dataset did not fit a stationary process.

Wavelet analyses using Morlet wavelet were performed with both centralized influenza and RSV positives to check stationarities. The intensities of virus seasonality were quantified by the power value at the 1-year period in the global wavelet spectrum.

A state-space model was then used to investigate the effect of climate variables. This model provides a unified framework for modelling time series data such as climate and surveillance data [56]. Briefly, the model is described as follows:

$$y_{t}=Z_{t}\;\alpha_{t}+\epsilon_{t}$$ (1)

$$\alpha_{t+1}=T_{t}\alpha_{t}+\omega_{t}$$ (2)

where y_t_ is the observation and *α*_*t*_ is an unobservable state of the system at time *t*. To standardize the annual distribution of virus positives, the proportions of annual influenza A, B, and RSV positives were set as dependent variables, thus following a normal distribution. We first set a local trend model as the baseline model, then applied a dynamic linear regression model with five climate variables: mean temperature, RH, SH, precipitation, and rainy days. Akaike’s Information Criterion (AIC) was estimated with log-likelihood, as follows:

AIC=−2×loglikelihood+2(V+P)⁡ (3)

where V is the number of state variables and P is the number of parameters.

The models’ fitted values were plotted against the observed data to visually assess model fit. Lastly, we calculated the percentage of improved model fit, as follows:

$$\%\;improved\;model\;fit=\frac{\left(Final\;model\;AIC–Baseline\;model\;AIC\right)}{Baseline\;model\;AIC}\times 100\%$$ (4)

Because the baseline model includes only non-climate variables, this percentage indicates by how much the included climate variable has improved the model fit. For result interpretation, the estimated time dependent coefficients from the univariate models were plotted.

A geographical map was drawn using ArcGIS 10.3 (ESRI, Redlands, CA, USA). All statistical analyses were done using R 3.2.3 [57]. KFAS [58] and biwavelet [59] packages were respectively used for dynamic linear regression model and wavelet analysis. A *p*-value of <0.05 was considered statistically significant.

### Ethics Statements

A written informed consent was obtained from either patients or a parent or guardian of any child participant in the Philippines sites. All data was anonymized. An approval for the study design and protocol was obtained from Research Institute of Tropical Medicine (RITM) Institutional Review Board.

## Supporting Information

S1 Fig Weekly trends of climate variables in Eastern Visayas, Baguio, and Okinawa, from January 2010 to December 2012: (from top) mean temperature, specific humidity, relative humidity, rainfall amount, and number of rainy days. Shaded areas show the interquartile ranges.
(TIF)

S2 Fig Coefficients of climate variables estimated with state-space model for influenza. (TIF)

S3 Fig Comparison of time series of rapid test kit usage (top) and RT-PCR confirmed influenza A (middle) and B (bottom). (TIF)

S1 Table Review of previous studies for influenza A and B in tropical countries (Categorized by temperature, humidity, and precipitation). (DOCX)

S2 Table Review of previous studies for RSV in tropical countries (Categorized by temperature, humidity, and precipitation). (DOCX)

S3 Table Model performance for influenza to model fit with climate variables in Eastern Visayas, Baguio, and Okinawa. (DOCX)

S4 Table Minimal dataset. (XLS)

S1 Text Formulae for calculating mean atmospheric pressure and specific humidity. (DOCX)
