# Supplementary figures and images for: Seasonality of Influenza and Respiratory Syncytial Viruses and the Effect of Climate Factors in Subtropical–Tropical Asia Using Influenza-Like Illness Surveillance Data, 2010 –2012
Source: PLoS One. 2016 Dec 21;11(12):e0167712. doi: 10.1371/journal.pone.0167712 (PMC5176282; doi:10.1371/journal.pone.0167712)

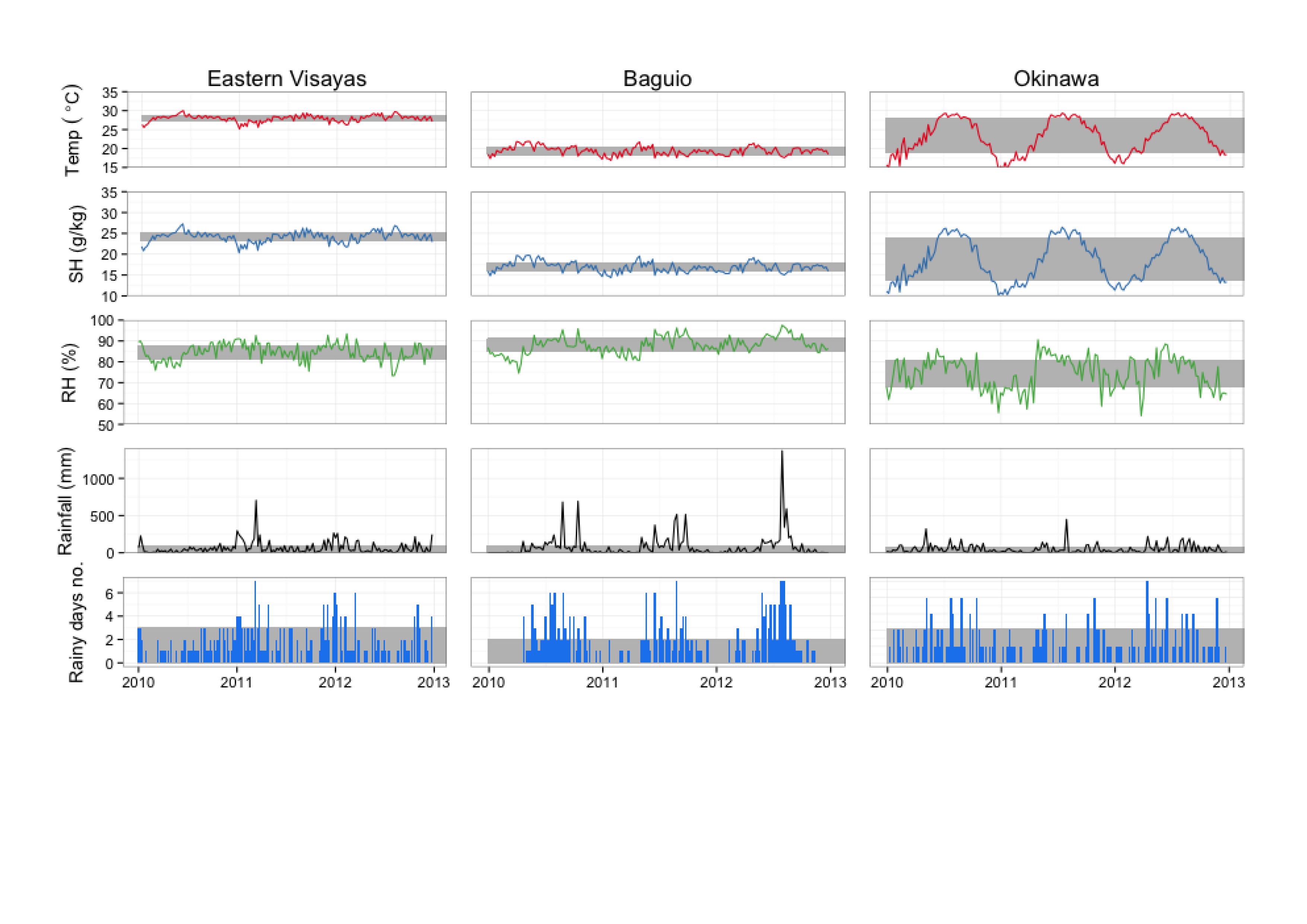

Supplement: S1 Fig — Shaded areas show the interquartile ranges. (TIF) [file pone.0167712.s001.tif]

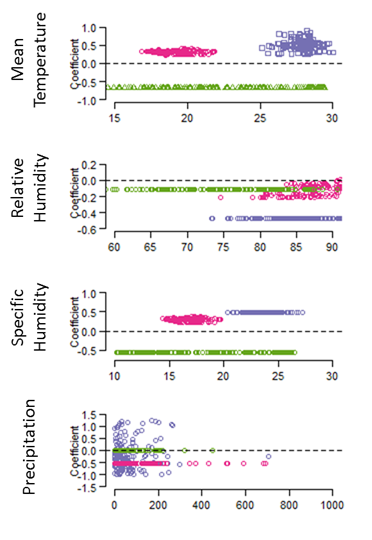

Supplement: S2 Fig — (TIF) [file pone.0167712.s002.tif]

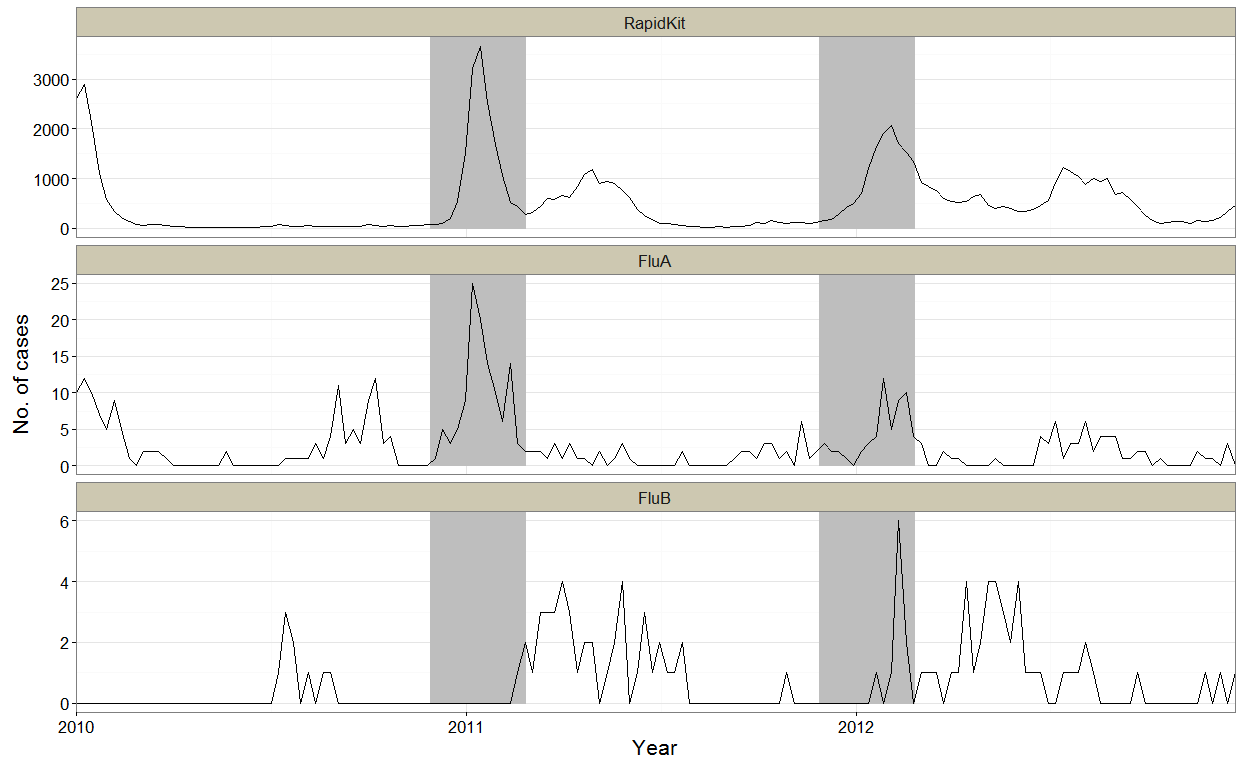

Supplement: S3 Fig — (TIF) [file pone.0167712.s003.tif]
