# Supplementary material for: Seasonality of Influenza and Respiratory Syncytial Viruses and the Effect of Climate Factors in Subtropical–Tropical Asia Using Influenza-Like Illness Surveillance Data, 2010 –2012
Source: PLoS One. 2016 Dec 21;11(12):e0167712. doi: 10.1371/journal.pone.0167712 (PMC5176282; doi:10.1371/journal.pone.0167712)
Supplement: S1 Table — (DOCX) [file pone.0167712.s004.docx]

**S1 Table. Review of previous studies for influenza A and B in tropical countries (Categorized by temperature, humidity, and precipitation).**

| **Variable group** | **Setting [Reference no.]** | **Latitude** | | **Study period** | | **Variable** | **Correlation type** | **Statistical method** |
| --- | --- | --- | --- | --- | --- | --- | --- | --- |
| Temperature | Singapore [1] | 1°18’N | | 2000–2007 | | aveT | Not found | Time series |
|  | Panama [2] | 8°58’N | | 2008–2013 | | aveT | Not found | Logistic regression |
|  | El Salvador [2] | 13°40’N | | 2008–2013 | | aveT | Negative | Logistic regression |
|  | Guatemala [2] | 14°38’N | | 2008–2013 | | aveT | Not found | Logistic regression |
|  | Hong Kong SAR [1, 3] | 22°18’N | | 2000–2007 | | aveT | Negative (flu B only) | Time series |
|  | Kolkata, India [4] | 22°39’N | | 2007–2008 | | minT, maxT | Positive | Pearson correlation test |
| Humidity | Singapore [1] | | 1°18’N | 2000–2007 | RH | | Negative | Time series |
|  | Panama [2] | | 8°58’N | 2008–2013 | SH | | Positive | Logistic regression |
|  | El Salvador [2] | | 13°40’N | 2008–2013 | SH | | Positive | Logistic regression |
|  | Guatemala [2] | | 14°38’N | 2008–2013 | SH | | Negative | Logistic regression |
|  | Hong Kong SAR [1, 3] | | 22°18’N | 2000–2007  1997–2006 | RH | | Positive (flu A only) | Time series |
|  | Kolkata, India [4] | | 22°39’N | 2007–2008 | RH | | Positive | Pearson correlation test |

| **Variable group** | **Setting** | **Latitude** | **Study period** | **Variable** | **Correlation type** | **Statistical method** |
| --- | --- | --- | --- | --- | --- | --- |
| Precipitation | Singapore [5] | 1°18’N | 1990–1994 | Rainfall | Positive (flu B only) | Time series |
|  | Singapore [1] | 1°18’N | 2000–2007 | Rainfall | Not found | Time series |
|  | Fortaleza, northeast Brazil [6] | 4°S | 2001–2007 | Rainy season | Positive | Inspect graph only |
|  | Indonesia [7] | 6°10.5’S | 2003–2007 | Mean rainfall | Positive (flu A only) | Pearson correlation test |
|  | Cote d’Ivoire [8] | 6°51’N | 2003–2010 | Rainy season | Positive | Inspect graph only |
|  | Thailand [9] | 6°– 22°N | 2004–2010 | Rainy season | Positive | Inspect graph only |
|  | Panama [2] | 8°58’N | 2008–2013 | Rainfall | Positive | Logistic regression |
|  | Cambodia [10] | 11°33’N | 2006–2008 | Rainy season | Positive | Inspect graph only |
|  | El Salvador [2] | 13°40’N | 2008–2013 | Rainfall | Not found | Logistic regression |
|  | Guatemala [2] | 14°38’N | 2008–2013 | Rainfall | Positive | Logistic regression |
|  | Senegal [11] | 14°40’N | 1996–2009 | Rainy season | Positive | Inspect graph only |
|  | Myanmar [12] | 19°45’N | 2006–2007 | Rainy season | Positive | Inspect graph only |
|  | Hong Kong SAR [1] | 22°18’N | 2000–2007 | Rainfall | Not found | Time series |
|  | Kolkata, India [4] | 22°39’N | 2007–2008 | Rainfall | Positive | Pearson correlation test |
|  | Bangladesh [13] | 23°42’N | 2007–2008 | Rainy season | Positive | Correlation test |

*Abbreviations: aveT, average temperature; minT, minimum temperature; maxT, maximum temperature; RH, relative humidity; SH, specific humidity.

**Number in parentheses[ ] indicates reference number of the cited article.

**References:**

1. Tang JW, Lai FY, Nymadawa P, Deng YM, Ratnamohan M, Petric M, et al. Comparison of the incidence of influenza in relation to climate factors during 2000–2007 in five countries. Journal of Medical Virology. 2010;82(11):1958-65.

2. Soebiyanto RP, Clara W, Jara J, Castillo L, Sorto OR, Marinero S, et al. The Role of Temperature and Humidity on Seasonal Influenza in Tropical Areas: Guatemala, El Salvador and Panama, 2008–2013. PLoS One. 2014;9(6):e100659.

3. Chan PKS, Mok HY, Lee TC, Chu IMT, Lam W-Y, Sung JJY. Seasonal influenza activity in Hong Kong and its association with meteorological variations. Journal of Medical Virology. 2009;81(10):1797-806.

4. Agrawal AS, Sarkar M, Chakrabarti S, Rajendran K, Kaur H, Mishra AC, et al. Comparative evaluation of real-time PCR and conventional RT-PCR during a 2 year surveillance for influenza and respiratory syncytial virus among children with acute respiratory infections in Kolkata, India, reveals a distinct seasonality of infection. Journal of Medical Microbiology. 2009;58(12):1616-22.

5. Chew FT, Doraisingham S, Ling AE, Kumarasinghe G, Lee BW. Seasonal trends of viral respiratory tract infections in the tropics. Epidemiology and Infection. 1998;121(01):121-8.

6. Moura FEA, Perdigao ACB, Siqueira MM. Seasonality of influenza in the tropics: A distinct pattern in Northeastern Brazil. American Journal of Tropical Medicine and Hygiene. 2009;81(1):180-3.

7. Kosasih H, Roselinda, Nurhayati, Klimov A, Xiyan X, Lindstrom S, et al. Surveillance of Influenza in Indonesia, 2003–2007. Influenza and Other Respiratory Viruses. 2013;7(3):312-20.

8. Kadjo HA, Ekaza E, Coulibaly D, Kouassi DP, Nzussouo NT, Kouakou B, et al. Sentinel surveillance for influenza and other respiratory viruses in Côte d’Ivoire, 2003–2010. Influenza and Other Respiratory Viruses. 2013;7(3):296-303.

9. Chittaganpitch M, Supawat K, Olsen SJ, Waicharoen S, Patthamadilok S, Yingyong T, et al. Influenza viruses in Thailand: 7 years of sentinel surveillance data, 2004–2010. Influenza and Other Respiratory Viruses. 2012;6(4):276-83.

10. Mardy S, Ly S, Heng S, Vong S, Huch C, Nora C, et al. Influenza activity in Cambodia during 2006-2008. BMC Infectious Diseases. 2009;9(1):168. PubMed PMID: doi:10.1186/1471-2334-9-168.

11. Niang MN, Dosseh A, Ndiaye K, Sagna M, Gregory V, Goudiaby D, et al. Sentinel Surveillance for Influenza in Senegal, 1996–2009. Journal of Infectious Diseases. 2012;206(suppl 1):S129-S35.

12. Dapat C, Saito R, Kyaw Y, Naito M, Hasegawa G, Suzuki Y, et al. Epidemiology of human influenza A and B viruses in Myanmar from 2005 to 2007. Intervirology. 2009;52(6):310-20.

13. Zaman RU, Alamgir ASM, Rahman M, Azziz-Baumgartner E, Gurley ES, Sharker MAY, et al. Influenza in outpatient ILI case-patients in national hospital-based surveillance, Bangladesh, 2007–2008. PLoS ONE. 2009;4(12):e8452.
