# Supplementary material for: Seasonality of Influenza and Respiratory Syncytial Viruses and the Effect of Climate Factors in Subtropical–Tropical Asia Using Influenza-Like Illness Surveillance Data, 2010 –2012
Source: PLoS One. 2016 Dec 21;11(12):e0167712. doi: 10.1371/journal.pone.0167712 (PMC5176282; doi:10.1371/journal.pone.0167712)
Supplement: S2 Table — (DOCX) [file pone.0167712.s005.docx]

**S2 Table.** **Review of previous studies for RSV in tropical countries (Categorized by temperature, humidity, and precipitation).**

| **Variable group** | **Setting** | **Latitude** | **Study period** | **Variable** | **Correlation type** | **Statistical method** |
| --- | --- | --- | --- | --- | --- | --- |
| Temperature | Singapore [1] | 1°18’N | 2000–2007 | aveT, maxT | Positive | Time series |
|  | Kuala Lumpur, Malaysia [2] | 3°08’N | 1982–2008 | aveT | Negative | Multiple regression |
|  | Lombok island, Indonesia [3] | 8°–9°S | 2000–2002 | aveT | Positive | Negative binomial regression |
|  | Hong Kong SAR [4] | 22°18’N | 1993–1997 | aveT | Positive | Multivariate regression |
|  | Kolkata, India [5] | 22°39’N | 2007–2008 | minT, maxT | Negative | Pearson correlation test |
|  | Sao Paolo, southeast Brazil [6] | 23.4°S | 1996–2010 | minT | Negative | Fourier analysis and Spearman’s correlation test |
|  | Netherlands [7] | 52°22’N | 1998–2005 | minT | Negative | General linear methods |
| Humidity | Singapore [1] | 1°18’N | 2000–2007 | RH | Negative | Time series |
|  | Kuala Lumpur, Malaysia [2] | 3°08’N | 1982–2008 | RH | Negative | Multiple regression |
|  | Lombok island, Indonesia [3] | 8°–9°S | 2000–2002 | RH | Positive | Negative binomial regression |
|  | Hong Kong SAR [4] | 22°18’N | 1993–1997 | RH | Positive | Multivariate regression |
|  | Netherlands [7] | 52°22’N | 1998–2005 | RH | Positive | General linear methods |
| Precipitation | Kuala Lumpur, Malaysia [2] | 3°08’N | 1982–2008 | Rain day | Positive | Multiple regression |
|  | Fortaleza, Northeast Brazil [8] | 4°S | 2004–2008 | Rainy season | Positive | Inspect graph only |
|  | Lombok island, Indonesia [3] | 8°–9°S | 2000–2002 | Occurrence of rain | Positive | Negative binomial regression |
|  | Kolkata, India [5] | 22°39’N | 2007–2008 | Rainfall | Negative | Pearson correlation test |
|  | Sao Paolo, southeast Brazil [6] | 23.4°S | 1996–2010 | Rainfall | Negative | Fourier analysis and Spearman’s correlation test |

Abbreviations: aveT, average temperature; mint, minimum temperature; maxT, maximum temperature; RH, relative humidity.

Number in parentheses[ ] indicates reference number of the cited article

**References:**

1. Chew FT, Doraisingham S, Ling AE, Kumarasinghe G, Lee BW. Seasonal trends of viral respiratory tract infections in the tropics. Epidemiology and Infection. 1998;121(01):121-8. doi: doi:10.1017/S0950268898008905.

2. Khor C-S, Sam I-C, Hooi P-S, Quek K-F, Chan Y-F. Epidemiology and seasonality of respiratory viral infections in hospitalized children in Kuala Lumpur, Malaysia: a retrospective study of 27 years. BMC Pediatrics. 2012;12(1):32. PubMed PMID: doi:10.1186/1471-2431-12-32.

3. Omer SB, Sutanto A, Sarwo H, Linehan M, Djelantik IGG, Mercer D, et al. Climatic, temporal, and geographic characteristics of respiratory syncytial virus disease in a tropical island population. Epidemiology and Infection. 2008;136:1319-27.

4. Chan PK, Sung RY, Fung KS, Hui M, Chik KW, Adeyemi-Doro FA, et al. Epidemiology of respiratory syncytial virus infection among paediatric patients in Hong Kong: seasonality and disease impact. Epidemiology and Infection. 1999;123(2):257-62. PubMed PMID: PMC2810757.

5. Agrawal AS, Sarkar M, Chakrabarti S, Rajendran K, Kaur H, Mishra AC, et al. Comparative evaluation of real-time PCR and conventional RT-PCR during a 2 year surveillance for influenza and respiratory syncytial virus among children with acute respiratory infections in Kolkata, India, reveals a distinct seasonality of infection. Journal of Medical Microbiology. 2009;58(12):1616-22. doi: 10.1099/jmm.0.011304-0.

6. Paiva TM, Ishida MA, Benega MA, Constantino CRA, Silva DBB, Santos KCO, et al. Shift in the timing of respiratory syncytial virus circulation in a subtropical megalopolis: Implications for immunoprophylaxis. Journal of Medical Virology. 2012;84(11):1825-30.

7. Meerhoff T, Paget J, Kimpen J, Schellevis F. Variation of Respiratory Syncytial Virus and the Relation With Meteorological Factors in Different Winter Seasons. Pediatric Infectious Disease Journal. 2009;28(10):860-6. doi: 10.1097/INF.0b013e3181a3e949. PubMed PMID: WOS:000270407800002.

8. Moura FEA, Perdigão ACB, Ribeiro JF, Florêncio CMGD, Oliveira FMS, Pereira SAR, et al. Respiratory syncytial virus epidemic periods in an equatorial city of Brazil. Influenza and Other Respiratory Viruses. 2013;7(6):1128-35.
