# Supplementary material for: Seasonality of Influenza and Respiratory Syncytial Viruses and the Effect of Climate Factors in Subtropical–Tropical Asia Using Influenza-Like Illness Surveillance Data, 2010 –2012
Source: PLoS One. 2016 Dec 21;11(12):e0167712. doi: 10.1371/journal.pone.0167712 (PMC5176282; doi:10.1371/journal.pone.0167712)
Supplement: S3 Table — (DOCX) [file pone.0167712.s006.docx]

**S3 Table Model performance for influenza to fit with climate variables in Eastern Visayas, Baguio, and Okinawa.**

|  | Model | **Eastern Visayas** | | **Baguio** | | **Okinawa** | |
| --- | --- | --- | --- | --- | --- | --- | --- |
|  |  | AIC | Improved model fit (%) | AIC | Improved model fit (%) | AIC | Improved model fit (%) |
| Influenza | Local trend | −770.2 |  | −646.4 |  | −597.8 |  |
|  | Mean temperature | −761.0 | 1.2 | −641.1 | 0.8 | −591.0 | 1.1 |
|  | RH | −763.9 | 0.8 | −643.4 | 0.4 | −595.1 | 0.5 |
|  | SH | −765.1 | 0.7 | −641.6 | 0.7 | −592.0 | 1.0 |
|  | Rainfall | −763.9 | 0.8 | −632.2 | 2.2 | −596.3 | 0.2 |
|  | Rainy days ^a^ | −799.1 | -3.8 | −659.8 | -2.1 | −663.6 | -11.0 |

Abbreviations: AIC: Akaike Information Criterion, RH: relative humidity, SH: specific humidity.

^a^ Rainy day is defined as the number of rainy days in one week with > 10 mm precipitation.
