# Supplementary material for: Seasonality of Influenza and Respiratory Syncytial Viruses and the Effect of Climate Factors in Subtropical–Tropical Asia Using Influenza-Like Illness Surveillance Data, 2010 –2012
Source: PLoS One. 2016 Dec 21;11(12):e0167712. doi: 10.1371/journal.pone.0167712 (PMC5176282; doi:10.1371/journal.pone.0167712)
Supplement: S1 Text — (DOCX) [file pone.0167712.s008.docx]

Supporting Information for: "Seasonality of influenza and respiratory syncytial viruses and the effect of climate factors in subtropical–tropical Asia using influenza-like illness surveillance data, 2010–2012"

**S1 Text. Formulae for calculating mean atmospheric pressure and specific humidity.**

Vapour pressure, another measure of absolute humidity, was also evaluated in this study. However, we chose to include only SH because the atmospheric pressure component is included in the formulae, unlike vapour pressure. We also investigated the effect of altitude; thus, we believe SH is a more suitable variable for this study.

Owing to the lack of atmospheric pressure data in Baguio City (BC), the barometric formula was used to obtain mean atmospheric pressure, which was then applied to the weekly SH value calculations. We assumed that the atmospheric pressure did not change significantly from the mean during the study period. We also checked the difference in the current atmospheric pressure data obtained over a 1-month period and verified that this difference was less than 1%.

1. Barometric formula

$$p = p_{0} . \left( 1 - \frac{L . h}{T_{0}} \right)^{\frac{g. M}{R. L}}$$

where, p = atmospheric pressure to be calculated

p_0_ = standard atmospheric pressure at sea level (101325 Pa)

L = temperature lapse rate (0.0065 K/m)

h = altitude of BC above sea level (1500 m)

g = Earth’s surface gravitational acceleration (9.81 m/s^2^)

M = molar mass of dry air (0.029 kg/mol)

R = universal gas constant (8.314 J/mol K)

T_0_ = standard temperature at sea level (288.15 K)

1. The following two-step process was used to calculate SH:

(1) $\mathrm{mixing} ratio (MR) = 0.622 x \frac{e_{s}}{p - e_{s}}$

(2) $SH = \frac{MR}{1 + MR}$

For (1), $e_{s}$is estimated using the following formulae:

$$e_{s}\left( T \right)=e_{s}\left( To \right) \times e^{[\frac{L}{Rv}\left( \frac{1}{To}-\frac{1}{T} \right)]}$$

where,

e_s_ (T) = saturation vapour pressure at temperature T

e_s_ (T_0_) = saturation vapour pressure at T_0_ (6.11 mb)

T_0_ = reference temperature (273.15 K)

L = latent heat of evaporation for water (2.5 x 10^6^ J/kg)

R_v_ = gas constant for water vapour (461.5 J/kg)

T = temperature in Kelvin
